# Modification of DNA structure by reactive nitrogen species as a result of 2-methoxyestradiol–induced neuronal nitric oxide synthase uncoupling in metastatic osteosarcoma cells

**DOI:** 10.1016/j.redox.2020.101522

**Published:** 2020-03-28

**Authors:** Magdalena Gorska-Ponikowska, Agata Ploska, Dagmara Jacewicz, Michal Szkatula, Giampaolo Barone, Giosuè Lo Bosco, Fabrizio Lo Celso, Aleksandra M Dabrowska, Alicja Kuban-Jankowska, Monika Gorzynik-Debicka, Narcyz Knap, Lech Chmurzynski, Lawrence Wawrzyniec Dobrucki, Leszek Kalinowski, Michal Wozniak

**Affiliations:** a Department of Medical Chemistry, Medical University of Gdansk, 1 Debinki St, 80-211, Gdansk, Poland; b Euro-Mediterranean Institute of Science and Technology, Palermo, Italy; c Department of Biophysics, Institute of Biomaterials and Biomolecular Systems, University of Stuttgart, Stuttgart, Germany; d Department of Medical Laboratory Diagnostics, Medical University of Gdansk, Gdansk, Poland; e Biobanking and Biomolecular Resources Research Infrastructure Poland (BBMRI.PL), Gdansk, Poland; f Department of General and Inorganic Chemistry, University of Gdansk, Gdansk, Poland; g Department of Biological, Chemical and Pharmaceutical Sciences and Technologies, University of Palermo,Palermo, Italy; h Department of Mathematics and Computer Science, University of Palermo, Palermo, Italy; i Department of Physics and Chemistry "Emilio Segrè", University of Palermo, Palermo, Italy; j Department of Bioinorganic Chemistry University of Gdansk, Gdansk, Poland; k Department of Bioengineering, University of Illinois at Urbana-Champaign, Urbana, IL, USA; l Beckman Institute for Advanced Science and Technology, Urbana, IL, USA

## Abstract

2-methoxyestradiol (2-ME) is a physiological anticancer compound, metabolite of 17β-estradiol. Previously, our group evidenced that from mechanistic point of view one of anticancer mechanisms of action of 2-ME is specific induction and nuclear hijacking of neuronal nitric oxide synthase (nNOS), resulting in local generation of nitro-oxidative stress and finally, cancer cell death.

The current study aims to establish the substantial mechanism of generation of reactive nitrogen species by 2-ME. We further achieved to identify the specific reactive nitrogen species involved in DNA-damaging mechanism of 2-ME.

The study was performed using metastatic osteosarcoma 143B cells. We detected the release of biologically active (free) nitric oxide (^•^NO) with concurrent measurements of peroxynitrite (ONOO^−^) in real time in a single cell of 143B cell line by using ^•^NO/ONOO^−^ sensitive microsensors after stimulation with calcium ionophore. Detection of nitrogen dioxide (^•^NO_2_) and determination of chemical rate constants were carried out by a stopped-flow technique. The affinity of reactive nitrogen species toward the guanine base of DNA was evaluated by density functional theory calculations. Expression and localization of nuclear factor NF-kB was determined using imaging cytometry, while cell viability assay was evaluated by MTT assay.

Herein, we presented that 2-ME triggers pro-apoptotic signalling cascade by increasing cellular reactive nitrogen species overproduction – a result of enzymatic uncoupling of increased nNOS protein levels. In particular, we proved that ONOO^−^ and ^•^NO_2_ directly formed from peroxynitrous acid (ONOOH) and/or by auto-oxidation of ^•^NO, are inducers of DNA damage in anticancer mechanism of 2-ME. Specifically, the affinity of reactive nitrogen species toward the guanine base of DNA, evaluated by density functional theory calculations, decreased in the order: ONOOH > ONOO^−^ > ^•^NO_2_ > ^•^NO.

Therefore, we propose to consider the specific inducers of nNOS as an effective tool in the field of chemotherapy.

## 1 Introduction

2-methoxyestradiol (2-ME) is a physiological metabolite of 17β-estradiol synthesized by hydroxylation and subsequent *O*-methylation at the 2-position [[1], [2], [3]]. Serum levels of 2-ME range from 30 pM in men to as much as over 30 nM in pregnant women [[1], [2], [3]]; while, pharmacologically active plasma concentrations are equaled to μM concentrations [[1], [2], [3]].

2-ME is currently recognized as a potent inhibitor of angiogenesis and tumour growth. It has been evaluated in phase I/II clinical trials as a therapeutic for several types of cancer [[4], [5], [6], [7]]. While the molecular signalling mechanisms of 2-ME remain unclear, various possibilities have been examined. One study proposed that 2-ME combines with tubulin at or near the colchicine-binding site, thus inhibiting the polymerization of tubulin and resulting in mitotic arrest and apoptosis [8]. Other studies suggested possible inhibition of the pro-angiogenic hypoxia-inducible factor-1α, upregulation of p53 [9,10], or induction of oxidative stress [11].

Our team previously evidenced that from mechanistic point of view 2-ME selectively induces expression and nuclear translocation of neuronal nitric oxide synthase (nNOS), resulting in cancer cell death [[12], [13], [14]]. We further evaluated that 2-ME-cancer cell death is strictly associated with DNA damage and genomic instability due to nuclear localization of nNOS [12]. Nuclear nNOS was also strictly associated with regulation of mitochondrial biogenesis by 2-ME [14]. We established the metastatic osteosarcoma (OS 143B) cells as the convenient model for investigating 2-ME mode of action [[12], [13], [14]]. However, the exact reactive nitrogen species (RNS) responsible for 2-ME-mediated DNA damage still remain to be elucidated.

Most studies assume that generally nitric oxide synthase isoforms (NOSs) synthesize ^•^NO. However, it has to be taken into consideration that dysregulated NOSs may become a source of superoxide (O_2_^•–^) because of an enzymatic “uncoupling” of l-arginine oxidation and oxygen reduction by the oxygenase and reductase domains of NOSs, respectively [[15], [16], [17]]. Therefore, as documented accurately, NOSs has two activities: nitric oxide (^•^NO) or superoxide (O_2_^•−^)/peroxynitrite (ONOO^−^) synthesis [[15], [16], [17], [18], [19]]. Indeed, an excess of intracellular nitro-oxidative stress may result in cell damage and finally, cell death [[20], [21], [22]]. NO and even more probably, its secondary reactive nitrogen intermediates, such as ONOO^−^ or nitrogen dioxide (^•^NO_2_) are extremely harmful to the cells, including cancer cells [[20], [21], [22]].

Notably, for all three isoforms of NOS, endothelial (eNOS), inducible (iNOS) and nNOS, the catalytic cycle involves the cofactor tetrahydrobiopterin (BH_4_) donating electrons to the NOS Fe^2+^-O_2_ complex initiating arginine oxidation. The ratio of [BH_4_] to its oxidation product [BH_2_] is critical since both bind to the active site with equal affinity. When tissue BH_4_:BH_2_ is low as found in patients with cancer and chronic inflammatory diseases, more O_2_^•–^/ONOO^−^ and less ^•^NO are generated [18,19].

Therefore, in the present study, we aim to investigate whether from mechanistic point of view anticancer activity of 2-ME in previously established OS 143B cell death model may rely on nNOS uncoupling. We further analyzed the specific RNS are responsible for 2-ME-induced DNA damage.

## 2 Materials and methods

### 2.1 Reagents

Tissue culture media, antibiotic cocktail, heat inactivated fetal bovine serum, *N*^ω^-Nitroarginine-2,4-l-diaminobutyric amide di(trifluoroacetate) salt (l-NDBA), lipopolysaccharide (LPS) and 2-ME were purchased from Sigma–Aldrich. Methyl 3-amino-2,3-dideoxy-α-d-*arabino*-hexopyranoside (AaraNH_2_), *cis*-[Cr(C_2_O_4_) (AaraNH_2_)(OH_2_)_2_]^+^ were synthesized according to the procedures described respectively [[22], [23], [24], [25], [26], [27]]. The final uptake product, *cis*-[Cr(C_2_O_4_)(L-L)(ONO_2_)]^−^, was characterized according to a procedure described previously [[22], [23], [24], [25], [26], [27]].

### 2.2 Cell line and culture conditions

The human OS 143B cell line (ATTC-8303) was purchased from Sigma–Aldrich (Soborg, Denmark) [[12], [13], [14]]. The cells were maintained in monolayer culture using EMEM supplemented with 10% heat inactivated FBS, 2 mM l-glutamine, penicillin (100 U/ml)/streptomycin (100 μg/ml) cocktail. Cells were cultured at 37 °C in a humidified atmosphere saturated with 5% CO_2_.

### 2.3 Cell treatment

The human OS 143B cells were treated with different concentrations of 2-ME for different times, depending on the design of the specific experiments [[12], [13], [14]]. In order to exclude potential influence of serum-derived hormones, all experiments were performed in EMEM supplemented only with antibiotic. The data were obtained from at least three independent experiments (N = 6 replicate cultures).

### 2.4 Measeurement of nitric oxide and peroxynitrite by porphyrinic ultramicrosensors

The concurrent measurements of ^•^NO and ONOO^−^ released by cells cultured on 6-well plates were taken with two porphyrinic microelectrodes combined into one working unit as described [15,28,29]. Each microsensor consisted of 5–7 carbon fibers (diameter 0.2 μm), which were electroplated with highly conductive polymeric porphyrins [nickel(II) tetrakis(3-methoxy-4-hydroxyphenyl) porphyrin for the NO-sensor and Mn(III)- [2,2]paracyclophanyl porphyrin for the ONOO^−^-sensor], which facilitate the electron transfer from ^•^NO or ONOO^−^ to the sensors. The microsensors were supercoated with polymers, Nafion for the NO-sensor and poly(4-vinylpyridine) for the ONOO^−^-sensor. These ultramicrosensors are highly sensitive to ^•^NO and ONOO^−^ with a detection limit of 1 nmol/L and a resolution time below 1 ms for each sensor. A three-electrode system, which includes a module of ^•^NO/ONOO^−^-sensitive sensors as a working electrodes, a counter platinum electrode, and a silver/silver chloride reference electrode, was used with a constant potential of 650 mV for ^•^NO detection and −450 mV for ONOO^−^ detection. The system was coupled with Gamry Reference 600 (Gamry Instruments, Warminster, PA, USA), multichannel potentiostat and a personal computer with electrochemical software (Gamry VFP600). The module of ^•^NO/ONOO^−^-sensitive ultramicrosensors was positioned with the help of a micromanipulator close to the cell culture surface (10 ± 1 μm). Before the experiment was started, the cells were pretreated for 30 min with or without 10 μM l-NDBA, nNOS specific antagonist, and the medium was changed to phenol red-free and serum free MEM medium To stimulate maximum nNOS-dependent ^•^NO and ONOO^−^ generations, the receptor independent nNOS agonist, calcium ionophore A23187 (1.0 μM), was injected with a micromanipulator-positioned nanoinjector, and the responses (current vs. time) were recorded continuously. The current was proportional to the local concentrations of ^•^NO and ONOO^−^ in the immediate vicinity of the sensor. Each sensor was calibrated with ^•^NO or ONOO^−^ standards, respectively.

### 2.5 Detection of nitrogen dioxide free radical

*Cell lysate preparation*. After treatment, both attached and detached cells were collected and centrifuged (200×*g*, for 5 min). The cell pellets were washed twice with PBS and then were resuspended in 3 ml of extraction buffer (150 mM NaCl, 5 mM EDTA, 1% Triton X-100, 10 mM Tris-HCl pH 7.4). Insoluble cellular debris was pelleted by centrifugation (500×*g*, for 10 min). Supernatants were then analyzed by a stopped-flow technique. As both O_2_^•–^ and ^•^NO are relatively more soluble in the hydrophobic phase of biological membranes, the lipid bilayers seem to be a preferential cellular compartment for the process of ^•^NO oxidation [20,21]. To release ^•^NO_2_ from the hydrophobic cores of the cell membranes, the ^•^NO_2_ detection method was modified via the addition of Triton X-100 to the extraction buffer. This approach significantly improved availability of ^•^NO_2_ for biosensor detection, allowing for a more accurate determination of the total amount of ^•^NO_2_ within the cell [30,31]. In order to exclude the possibility of artifactual detection of the ^•^NO_2_ being spontaneously generated from ^•^NO in a cell free system upon extraction, we performed a control experiment by saturating the Triton X-100 extraction buffer with gaseous ^•^NO. ^•^NO_2_ levels were then measured. The negative result of this experiment supports our hypothesis that this ^•^NO_2_ detection method is meaningful and applicable to biological systems [[22], [23], [24], [25], [26], [27]].

### 2.6 Kinetic analysis

Determination of chemical rate constants was carried out by a stopped-flow technique using the Applied Photophysics SX-17MV spectrophotometer. The observable rate constants were computed with “Glint” software [32]. A global analysis of the data acquired for 37 wavelengths, within the range of 340–700 nm at 10 nm increments, was performed for different reaction models.

### 2.7 ^•^NO_2_ binding buffer preparation and data analysis

A ^•^NO_2_ binding complex solution was prepared by combining 0.5 ml of 1.0 mM *cis*-[Cr(C_2_O_4_)(AaraNH_2_)(OH_2_)_2_]^+^, 2 ml of 200 mM Tris buffer (pH 7.4) and 2 ml of 2 M NaClO_4_. The temperature of the solution was maintained at 20.0 °C ± 0.1 °C. Calculations of ^•^NO_2_ concentration were performed with Origin 6.0 software (OriginLab Corp., USA), on the basis of absorbance variations at wavelength λ = 541 nm, applying the nonlinear least squares method [31]. A global analysis of the acquired data was performed for different reaction models.

### 2.8 NF-kB p65 immunostaining by imaging cytometry

The immunostaining was performed as previously described [12]. For NFkB p65 immunostaining, interphase nuclei were used. Briefly, after 2-, 6-, and 8-h treatments with 2-ME in the 96-well plate, 143B OS cells were fixed with 3.7% formaldehyde containing 0.1% Triton X-100 in PBS for 20 min. Subsequently, the cells were incubated with 1% bovine serum albumin (BSA) in PBST (phosphate buffered saline containing 0.25% Triton X-100) at room temperature for 30 min. After washing with PBST, the cells were incubated with mouse monoclonal antibodies against NFkB p65 (Abcam, Germany) (diluted 1:50 in PBST–BSA [PBST containing 1% BSA]) overnight at 4 °C. The next day, FITC-conjugated, secondary polyclonal rat antibodies against mouse IgG (BD Biosciences, Germany) (diluted 1:1000 in PBST–BSA) were added and incubated at room temperature for 1 h. Nuclei were visualized with Hoechst 33342. Digital cell images were captured with an In Cell Analyzer 2000 (GE Healthcare, UK) equipped with a high performance CCD camera. To analyze cellular nNOS content and localization, In Cell Analyzer software (In Cell Analyzer Investigator) was used. The fluorescence density was presented in relative fluorescence units (RFUs) [2].

### 2.9 Cell viability assay (MTT assay)

The MTT assay was performed as previously described [[12], [13], [14]]. The results were presented as a percentage of control (c). Each experiment was performed at least three times.

### 2.10 Density functional theory (*DFT) calculations*

The structures of the non-covalent complexes between ^•^NO, ^•^NO_2_, ONOO^−^, ONOOH and 2-deoxy-guanosine-5′-hydrogenphosphate (DGP), were investigated by DFT calculations, using the B97D [32] functional, which includes a correction for dispersion interactions, and the 6-31G(d,p) [33,34] basis set, with full geometry optimization. DGP was considered as a model of a guanine base of DNA, which is known to possess the lowest oxidation potential compared to the other DNA bases. The DNA affinity was estimated by the binding energy, through the following equation: ΔE = E(DGP-NOx)-E(DGP)-E(NOx), where NOx is NO, ^•^NO_2_, ONOO^−^ or ONOOH, DGP-NOx is the complex of DGP with NOx and E is the calculated SCF energy.

### 2.11 Statistical analysis

Data are presented as the mean ± SE values form at least three independent experiments. Data were analyzed using GraphPad Prism (GraphPad Software, Inc., version 6.03, La Jolla, CA, USA). Significant differences between groups were determined by One-way ANOVA combined with Dunnett's Multiple Comparison test or Student's *t*-test.

## 3 Results

### 3.1 2-ME enhances RNS production in osteosarcoma 143B cells

Previously we evidenced that 2-ME induced apoptosis in 143B OS cells not only at tested pharmacological relevant concentrations (10^−7^ M − 10^−5^ M), but also at physiological concentrations (10^−10^ M − 10^−8^ M) [12]. At least 10% of apoptotic 143B cells were observed in the presence of 2-ME ranging from concentrations of 10^−10^ M to 10^−6^ M [12,13]. We additionally proved that necrosis of 143B OS cells was observed only under pharmacological relevant concentrations (10^−6^ M and 10^−5^ M) of 2-ME [12,13].

We further determined that nNOS participates in the cell killing mechanism of 2-ME in the established OS cell death model [[12], [13], [14]]. Nω-Nitroarginine-2,4-l-diaminobutyric amide di(trifluoroacetate) salt (L-NDBA), selective inhibitor of nNOS, significantly increased the mean survival of the OS 143B cells in 2-ME-treated cultures [13]. Notably, 2-ME at both physiological and pharmacological relevant concentrations selectively induced nNOS overexpression what directly correlated with generation of ^•^NO in different cellular models, including OS [[12], [13], [14]]. Detected maximum peak of 2-ME-mediated-^•^NO release was time-dependent with within 6 and 8 h [12,13].

Herein, to further investigate whether after treatment with 2-ME the changes in NO result in NOS-dependent RNS, we measured the release of biologically active (free) ^•^NO with concurrent measurements of ONOO^−^ in real time in a single cell by ^•^NO/ONOO^−^-sensitive microsensors after stimulation with calcium ionophore. Basing on our above described studies [[12], [13], [14]], we chose representative pharmacological relevant concentration of 2-ME equaled 1 μM.

**Fig. 1:**
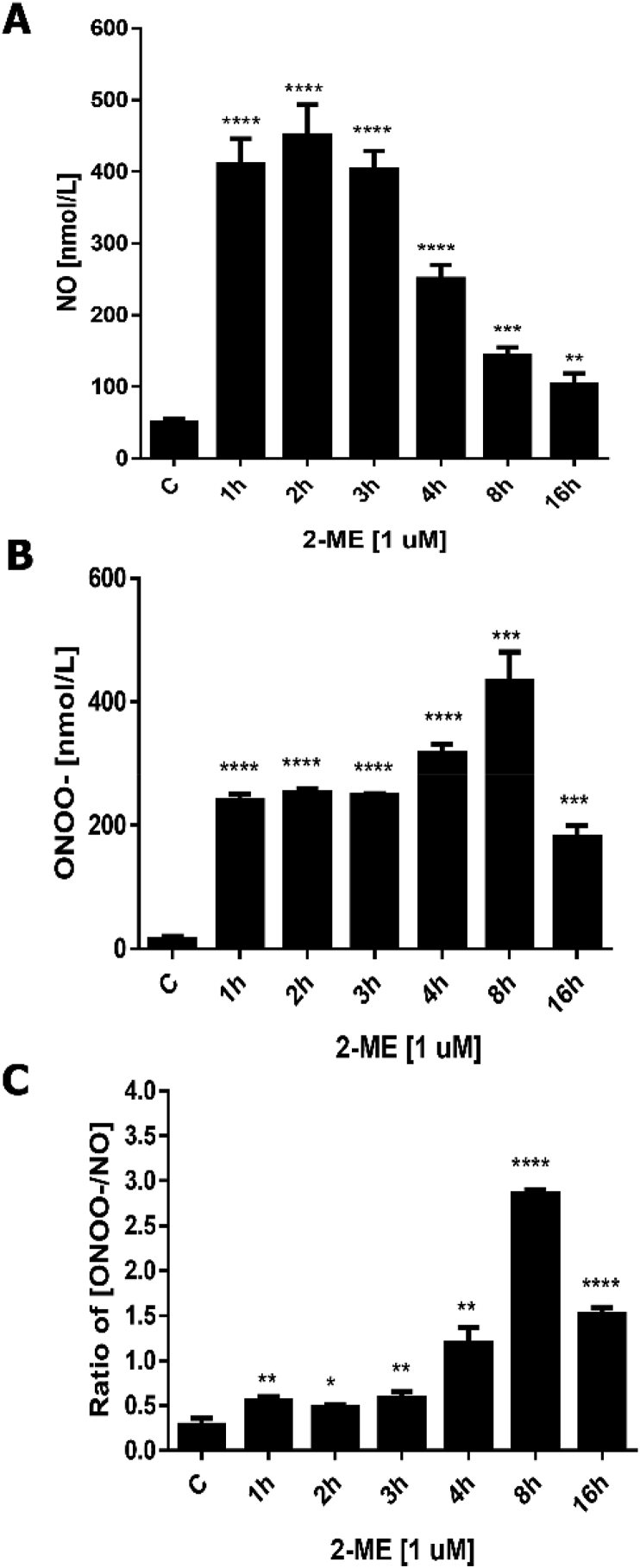
Effects of 2-ME treatment on RNS generation, *A.*^•^NO, *B.* ONOO^−^ and *C*. ONOO^−^/^•^NO ratio after NOS stimulation in cultured osteosarcoma 143B cells. ^•^NO and ONOO^−^ were detected simultaneously by the ^•^NO/ONOO^−^-sensitive specific microsensors in real time in the location with the highest concentrations, the surface of the cell membrane (in close vicinity of membrane-bound isoforms of NOS). ONOO^−^/^•^NO ratio is an indicator of nitro-oxidative stress level. At the end of the incubation periods with 1 μM 2-ME, ^•^NO and ONOO^−^ release was activated by the injection of calcium ionophore 1 μM A23187. Values are the mean ± SE of three independent experiments (N = 6 replicate cultures). The absence of error bar denotes a line thickness greater than the error. *P < 0.01; **P < 0.001; ***P < 0.0001; ****P < 0.00001 versus control cells (C).

The stimulation with calcium ionophore may produce a large increase in intracellular calcium levels, leading to full activation of available NOS isoforms in the cells, e.g. eNOS or nNOS (but not iNOS). Under certain conditions, NOS undergoes dysfunction (uncoupling) such as the enzyme produces not only ^•^NO but also superoxide (O_2_^•–^), that finally lead to the formation of ONOO^−^. Analysis of the time-dependent effect of the constant 2-ME concentration on stimulated ^•^NO and ONOO^−^ releases revealed the disparity in the maximal production of RNS (Fig. 1).

Similar to the flow cytometry analysis [12,13], the peak value of ^•^NO release from the cells was reached 2 h after the beginning of cell exposure to 2-ME (Fig. 1A). The peak value of ^•^NO production was about 8.5-fold higher than in the control (462 ± 21 vs. 55 ± 6). After this time-point production of ^•^NO gradually decreased with time-course of exposure of the cells to 2-ME. In contrast, the peak value of ONOO^−^ concentration was observed 8 h after treatment with 2-ME (Fig. 1B).

In our experimental model, the ratio of ONOO^−^ concentration to the NO concentration represents the index of RNS; higher [ONOO^−^/^•^NO] indicates weaker nNOS coupling and less bioavailability of NO within cells. As shown in Fig. 1C, the ratio of ONOO^−^ to ^•^NO maximal concentrations was significantly increased when the cells were exposed to 2-ME; the peak value was recorded at 8 h and was increased by almost 8.4-fold from 0.35 (in untreated cells) to 2.94.

### 3.2 nNOS is directly involved in 2-ME-mediated generation of intracellular NO and ONOO^−^

**Fig. 2:**
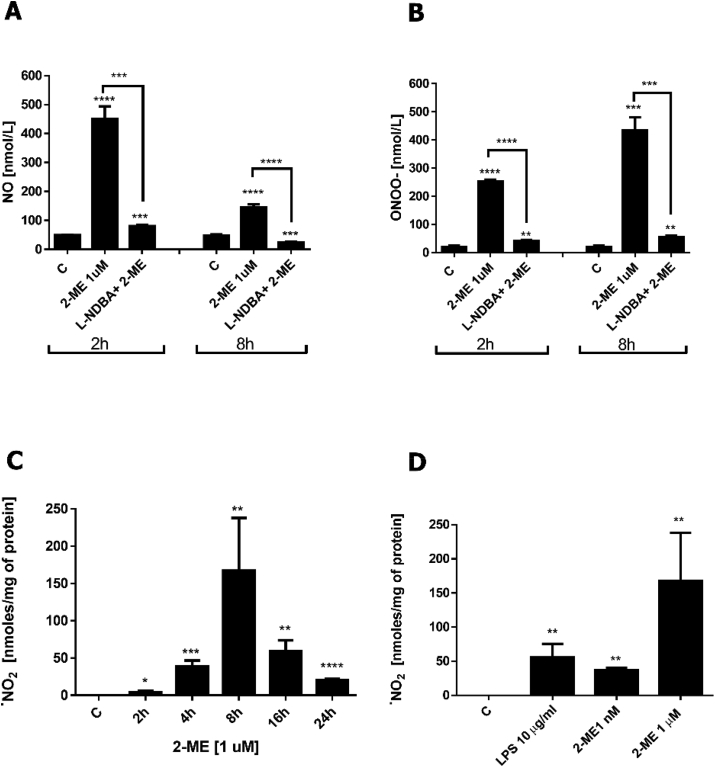
A,B. Effect of nNOS inhibition on 2-ME-induced changes in the generation of ^•^NO (*A*) and ONOO^−^ (*B*). At the end of 2-hr or 8-hr incubation periods with 1 μM 2-ME, ^•^NO and ONOO^−^ release was activated by the injection of calcium ionophore 1 μM A23187. Pretreatment with 10 μM L-NDBA significantly inhibited the release of RNS, ^•^NO and ONOO^−^, from osteosarcoma 143B cells treated with 2-ME. Values are the mean ± SE of three independent experiments (N = 6 replicate cultures). *P < 0.01; **P < 0.001; ***P < 0.0001; ****P < 0.00001 vs control cells (C). **C,D.** Impact of 2-ME on generation of ^•^NO_2_. A. Time-dependent generation of ^•^NO_2_ by 1 μM 2-ME. B. Comparison of generation of ^•^NO_2_ after 8 h incubation with 1 nM 2-ME, 1 μM 2-ME and 10 μg/ml LPS. Detection of ^•^NO_2_ was carried out using a specific molecular detector capable of selective ^•^NO_2_ binding, followed by a stopped-flow analysis. Values are the mean ± SE of three independent experiments (N = 6 replicate cultures). The absence of error bar denotes a line thickness greater than the error. *P < 0.01; **P < 0.001; ***P < 0.0001; ****P < 0.00001 vs control cells (C).

Using porphyrinic microsensors we further aimed to detect the levels of ^•^NO and ONOO^−^ after nNOS maximal stimulation with the receptor independent nNOS agonist, calcium ionophore A23187 (1 μM) in the presence of 1 μM 2-ME. We found that the release of ^•^NO and ONOO^−^ was related to nNOS activation, since nNOS inhibition by L-NDBA significantly reversed calcium ionophore-stimulated release of both ^•^NO and ONOO^−^ from 2-ME-treated cells (Fig. 2A and B). The obtained data confirmed 2-ME-induced nNOS uncoupling.

### 3.3 Treatment of 143B cells with 2-ME generates intracellular ^•^NO_2_

Due to the fact that ONOOH formed from ONOO^−^ may easily undergo hemolytic cleavage to hydroxyl and ^•^NO_2_ radicals [21], we further investigated the formation of this free radical by 2-ME using stopped-flow technique.

As detected with decreasing the levels of ^•^NO, we observed a simultaneous increase in production of ^•^NO_2_ (Fig. 2C). This trend was significantly pronounced after 4 h of 2-ME stimulation. The ^•^NO_2_ levels peaked at 8 h of treatment, reaching a value of 179 ± 28, ~179-fold higher compared to the unstimulated OS cells. However, the ^•^NO_2_ concentration considerably decreased after 16 h of treatment, which correlated with the massive cellular death observed in the samples. The data coincided with the peak ONOO^−^ generation. The peak value of ONOO^−^ concentration was observed 8 h after treatment with 2-ME (Fig. 1B), and it was at the time of the peak ^•^NO_2_ generation (Fig. 2C).

In order to give a new insight into plausible physiological anticancer activity of 2-ME [2,12,14], we next compared the effects of treatments with representative pharmacological (1 μM) and physiological relevant (1 nM) concentrations of 2-ME on ^•^NO_2_ generation (Fig. 2D). Interestingly, we also observed formation of ^•^NO_2_ after treatment with physiological relevant- 1 nM concentration of 2-ME. 2-ME at the physiological 1 nM concentration after 8 h of incubation increased level of ^•^NO_2_ by 36 fold (36.6 ± 2) (Fig. 2D). In order to obtain positive control, the data were correlated with production of ^•^NO_2_ generated after 8 h treatment with LPS (10 μg/ml). We observed ^•^NO_2_ production at the level of 55.4 nmol/mg ±11.4 as compared with the control cells (0 nmol/mg) (Fig. 2D).

### 3.4 Uncoupling of nNOS as the mechanistic mechanism of reactive nitrogen species generation by 2-ME

**Fig. 3:**
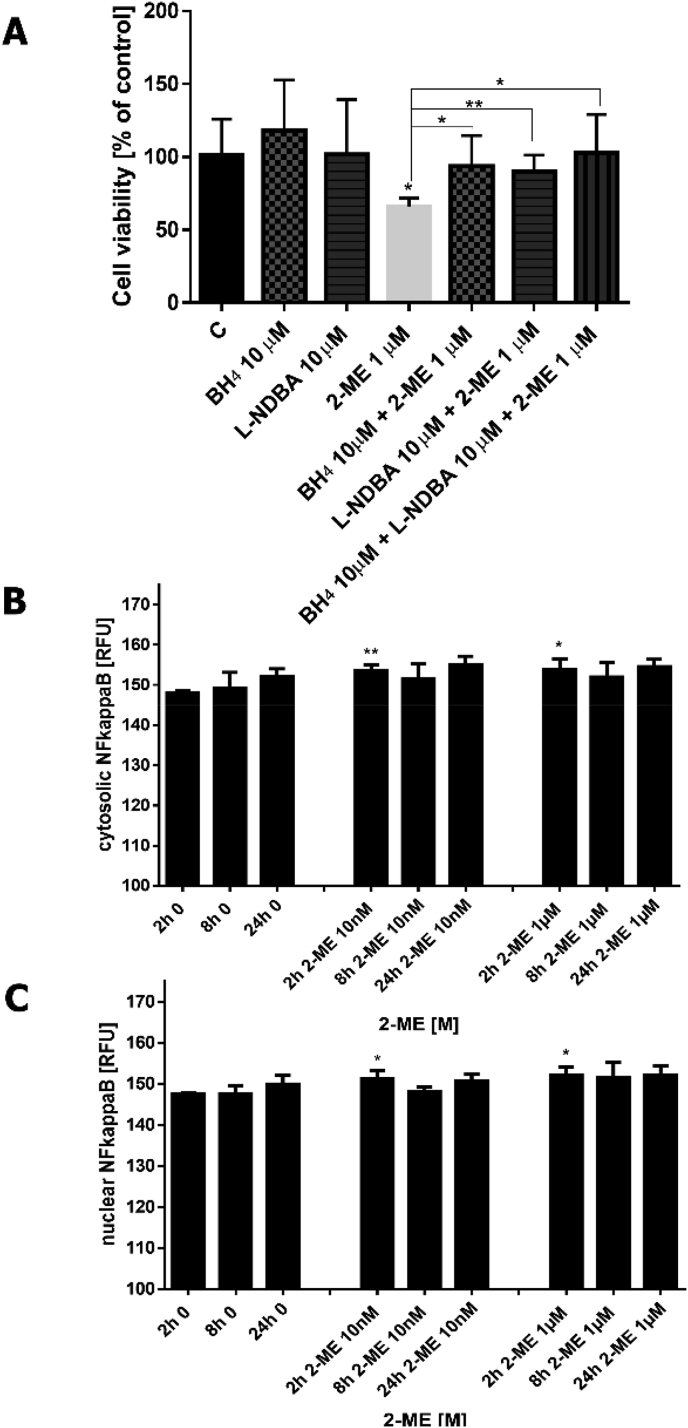
A. nNOS uncoupling induced by 2-ME. **B.** Impact of 2-ME on localization and expression of cytosolic and nuclear fractions of NF-kB p65. Localization of NF-kB p65 was determined by imaging cytometry after staining with specific anti–NF–kB antibody. The fluorescent signals were captured with an In Cell Analyzer 2000 (GE Healthcare, UK) equipped with a high-performance CCD camera. The fluorescence intensity is presented in relative fluorescence units (RFUs).Values are the mean ± SE of three independent experiments (N = 6 replicate cultures). The absence of error bar denotes a line thickness greater than the error. *P < 0.01; **P < 0.001; ***P < 0.0001; ****P < 0.00001 vs control cells (C).

We further investigated whether nNOS uncoupling due to enzyme overexpression and cofactor/coenzymes deficiency relies on anticancer mechanism of 2-ME. The OS 143B cells were treated with 10 μM BH_4_, 10 μM L-NDBA, 1 μM 2-ME separately or their combination for 48 h. Notably, addition of BH_4_ to 2-ME treated cells resulted in significant increased cell viability by 27% (Fig. 3A). Addition of L-NDBA, nNOS inhibitor, as expected decreased anticancer potential of 2-ME (Fig. 3A). Interestingly, treatment with both BH_4_ and L-NDBA totally reversed anti-proliferative effect of 2-ME (Fig. 3A).

### 3.5 Regulation of expression and intracellular localization of NF-κB by 2-ME

We further investigated the impact of 2-ME on localization of *NF-κB* p65 induced by 2-ME in time using imaging cytometry. OS 143B cells were treated with different concentrations of 2-ME – physiological relevant (10 nM), and pharmacological relevant (1 μM) for 2 h, 8 h, 24 h. As demonstrated we observed transient upregulation of both cytosolic and nuclear NF-*Κ*b p65 only after 2 h of incubation with all used concentrations of 2-ME (Fig. 3B and C). Notably, after 2 h, the level of NF-*Κ*b p65 in 2-ME-treated cells was decreased and comparable with control cells.

### 3.6 DNA is a target for reactive nitrogen species

**Fig. 4:**
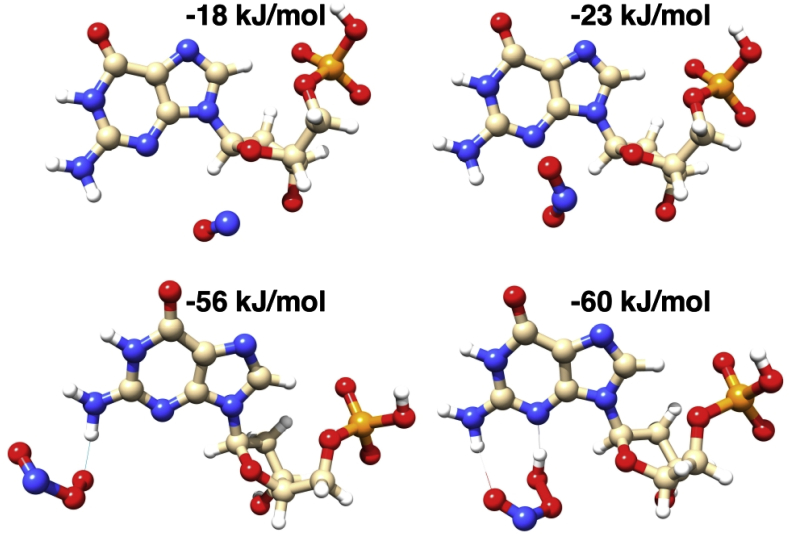
Optimized structures and binding energy of the non-covalent complexes between DGP and ^•^NO, ^•^NO_2_, ONOO^−^ and ONOOH, obtained by DFT calculations.

The final step of our study was to analyze what specific reactive nitrogen species are directly responsible for 2-ME-induced DNA damage in osteosarcoma cells. The results of the DFT calculations show that all considered RNS show affinity toward the guanine base (see Fig. 4). In particular, both ^•^NO and ^•^NO_2_ radicals loosely interact with the base in the region around the N3 atom, essentially by dispersion forces, with a binding energy of −18 kJ/mol and −23 kJ/mol, respectively. On the other hand, ONOO^−^ and ONOOH can interact with the guanine base also through hydrogen bonding: ONOO^−^ with the NH_2_ group and ONOOH with both NH_2_ and N3 groups, acting as both H-bond donor and acceptor. For this reason, the calculated binding energy is −56 kJ/mol and −60 kJ/mol for ONOO^−^ and ONOOH, respectively.

## 4 Discussion

2-ME is currently recognized as a potent anticancer agent [[1], [2], [3], [4], [5], [6], [7], [8], [9], [10], [11]]. It has been evaluated in several clinical trials as a therapeutic for several types of cancer [[4], [5], [6], [7]]. Unfortunately, the main limitation of usage of 2-ME in clinical practice is its poor pharmacokinetic profile [[4], [5], [6], [7]]. While the anticancer efficacy of 2-ME has been confirmed in various *in vitro* and *in vivo* studies [[1], [2], [3], [4], [5], [6], [7], [8], [9], [10], [11], [12], [13], [14]], the pleiotropic molecular mechanisms of 2-ME remain to be elucidated and explained.

Different studies indicated that anticancer mechanism of 2-ME relies on inhibition of the pro-angiogenic hypoxia-inducible factor-1α, upregulation of p53 [9,10], and/or induction of oxidative stress [11]. Moreover, 2-ME was previously reported to combine with tubulin at or near the colchicine-binding site, thus inhibiting the polymerization of tubulin and resulting in mitotic arrest and apoptosis [8]. However, the role of microtubule depolymerization in antitumor mechanism of 2-ME is controversial [35]. It was further suggested that the major mechanism of mitotic arrest at the pharmacological relevant concentrations of 2-ME is suppression of microtubule dynamics rather than microtubule depolymerization *per se* [35]. As established by our group, 2-ME perturbed the stability of microtubules analogously to taxol in OS 143B cells [36,37]. We then proposed that stability of microtubules controls the biogenesis of mitochondria [37]. Indeed, recently 2-ME was reported to affect mitochondrial biogenesis and mitochondria dynamics in OS 143B cells via its impact on microtubules [37] and selective ^•^NO generation due to nuclear hijacking of nNOS [14].

Despite its influence on mitochondria, the overexpression of nNOS is significantly correlated with induction of ^•^NO in nuclei of OS cells and closely associated with induction of DNA strand breaks and genomic instability [12]. Thus, induction of DNA damage by 2-ME may also be considered as one of its pleitropic anticancer mechanisms [12].

Therefore, herein, we aimed to investigate the substantial mechanism of 2-ME-induced overexpression of nNOS and further generation of specific RNS leading to DNA damage and cancer cell death.

NOS is a tightly coupled enzyme system that may be easily dysregulated under certain conditions such as perturbation in the availability of substrates or cofactors [21]. Notably, dysregulated NOS becomes a source of O_2_^•–^ because of an enzymatic “uncoupling” of l-arginine oxidation and oxygen reduction by the oxygenase and reductase domains of NOS, respectively [[15], [16], [17],^21^,^29^]. Any modification that partially uncouples NOS, with the formation of both O_2_^•–^ and ^•^NO, leads to the generation of ONOO^−^ and eventually of ^•^NO_2_, if ONOO^−^ is produced in excess [15,21,29]. The ability of NOS to produce O_2_^•–^ was first demonstrated for nNOS and then extended to eNOS [38,39].

Previously, our own studies revealed that eNOS is partially uncoupled in the normal human endothelium and that the degree of enzyme uncoupling varied in the endothelial cells depending on the ethnic group [15,29,40]. We were able to demonstrate using the tandem NO/O_2_^•–^/ONOO^−^-ultramicrosensors that eNOS activation in human endothelial cells implicates not only NO production, but also concomitant formation of both O_2_^•–^ and ONOO^−^.

Notably, the ratio of tetrahydrobiopterin: dihydrobiopterin (BH_4_:BH_2_) especially underlies the mechanism of NOS uncoupling [18,19]. The catalytic uncoupling also occurs at very low arginine or with increased levels of endogenous NOS inhibitors [41]. Herein, we propose that due to the induction of nNOS overexpression by 2-ME, the availability of BH_4_ is limited, what results in enzyme uncoupling. Indeed, we observed that addition of BH_4_ and selective nNOS inhibitor to 2-ME-treated cells reversed the anticancer effects of 2-ME, most probably due to nNOS re-coupling. It has to be emphasized that tumours may exert some level of just uncoupled NOSs [19]. The ratio of BH_4_:BH_2_ is found to be reduced in cancers including breast, colorectal or head tumours. It is also reduced in chronic inflammatory diseases [19]. As stated NOS uncoupling represents a crucial mechanism for cancer cell progression [19]. On the other side, as in case of 2-ME, uncoupled NOS produces RNS leading to cancer cell death [21].

The presented here results indicate that 2-ME cytotoxicity is not directly dependent on ^•^NO production, which reaches its peak as early as at 2 h of treatment, but rather on the production of the secondary ^•^NO-derived products, ONOO^−^ and finally formed ^•^NO_2_, whose levels peak soon before massive induction of apoptotic cell death. Indeed, we previously confirmed that, under physiological conditions, ONOO^−^ can be an efficient source of ^•^NO_2_ [17,22,26]. Because ONOO^−^ and ^•^NO_2_ are highly reactive and therefore unstable, it is difficult to analyze them within biological systems [21]. We have therefore developed novel, specific and highly efficient methods of the detection of ^•^NO, ONOO^−^ and ^•^NO_2_ released from the lipid bilayers of cells, making them available for biosensor measurements [[22], [23], [24], [25], [26], [27], [28], [29]]. The porphyrinic ^•^NO-ultramicrosensor detects only the net concentration of ^•^NO, i.e. ^•^NO that has not been consumed (yet) in fast chemical reaction and can freely diffuse to a target cell. This net NO concentration depends not only on the activity of NOS and the substrates, like oxygen and l-arginine, but also on the concentration of accumulated O_2_^•–^. The generation of O_2_^•–^ is calcium-dependent as is the production of ^•^NO by the NOS isoforms, i.e. nNOS and eNOS. Even though ^•^NO production can be high in cells, especially shortly after the injection of calcium ionophore, the concomitant progressively produced O_2_^•–^ rapidly combines (rate constant k = 9.6 × 10^9^ mol L^−1^ s^−1^) with ^•^NO in a diffusion-limited reaction to produce ONOO^−^.

It needs to be clearly emphasized that the fate of ONOO^−^ is highly dependent on its environmental conditions [21]. Its protonated form in acidic solutions (HOONO, pKa = 6.8) decomposes rapidly within <1 s [[42], [43], [44], [45], [46]]. The potential physiological generation of ONOO^−^/ONOOH results from the rapid reaction of O_2_^•−^ with ^•^NO, both known to be generated in significant amounts under certain pathophysiological conditions, including cancer. However, HOONO can react further with either ^•^NO or O_2_^•−^ to generate, among other things, ^•^NO_2_ [47,48]. In contrast to ONOO^−^, ONOOH can easily partition into the hydrophobic phase of lipid membranes [[47], [48], [49]]. Intracellular accumulation of ONOOH thus results in significant flux of this molecule across cellular membranes via passive or facilitated diffusion [[47], [48], [49]]. ONOOH may undergo homolytic cleavage to hydroxyl and ^•^NO_2_ radicals or alternatively heterolytic cleavage to a nitronium cation and a hydroxide anion [50,51].

Two of the products of the reaction between ^•^NO and O_2_^•–^, i.e. ONOO^−^ and ^•^NO_2,_ that were detected in the present study, are known to be cytotoxic, potentially inducing both apoptotic and necrotic pathways [21]. Both ONOO^−^ and ^•^NO_2_ can cause various kinds of cellular damage, including DNA and protein damage, and can activate cell death pathways [21]. Indeed, we previously evaluated that 2-ME-induced DNA damage and genomic instability are consequences of nuclear hijacking of nNOS and local induction of nitro-oxidative stress [12]. We observed single strand breaks as soon as after 2 h of incubation with both physiological and pharmacological relevant concentrations of 2-ME, and double strand breaks after 8 h of incubation with the compound [12].

Herein, we proved the affinity of RNS toward the guanine base of DNA as follows: ONOOH > ONOO^−^ > ^•^NO_2_ > ^•^NO. The biochemical nature of DNA damage mediated by RNS is associated with induction of genomic instability and cell death [52,53]. *In vitr*o studies with cellular models indicated that ^•^NO and its derivatives are able to induce direct- and mediated-genotoxic effects [[47], [48], [49]]. However, ^•^NO is not very reactive with DNA, but RNS formed by its reaction with oxygen radicals are potent DNA-damaging effects [52,53]. Several studies demonstrated that ^•^NO itself is not able to induce single strand breaks [52,53].

Therefore, nuclear hijacking of nNOS by 2-ME creates a perfect environment for formed RNS to interact with DNA. ONOO^−^ is the well-established DNA damaging factor [54,55]. DNA strand breaks induced by ONOO^−^ activate the repair enzyme, poly (ADP)-ribose (PARS). Excessive activation of PARS can lead to rapid consumption of NAD^+^ and ATP, and consequently to cell dysfunction and death by apoptosis or necrosis [56]. ONOO^−^ induces significantly more single strand breaks at acidic pH than at neutral or alkaline, what suggest that hydroxyl radical like intermediates or ONOOH are responsible for DNA damage. Indeed, herein we confirmed that ONOOH is the strongest factor in 2-ME-mediated DNA damage.

Our data indicate that beyond ONOO^−^ and its protonated form, also ^•^NO_2_ formed from protonated ONOO^−^ or by ^•^NO auto-oxidation seems to be additionally responsible for 2-ME-mediated DNA damage. Due to its electro-chemical nature, DNA is an ideal target for ^•^NO_2_ during nitro-oxidative stress [53]. Interestingly, ^•^NO tends to accumulate in the hydrophobic layers of biological membranes, where it often undergoes oxidation to its derivatives such as ONOO^−^ or ^•^NO_2_ (in case of the former, only under conditions of O_2_^•−^ bioavailability). Indeed, *in vitro* experiments presented that exposure to^•^ NO_2_ may cause DNA damage [[57], [58], [59]]. In addition, deleterious activity of 3-nitrotyrosine in the DNA model was indicated as an important implication of ^•^NO_2_ since aromatic nitration [60]. Indeed, in our previous studies we determined increased level of 3-nitrotyrosine after 8 h treatment with 2-ME [13] what correlates with the maximal level of generated ^•^NO_2_.

Interestingly, it is claimed that RNS are not able to induce double strand breaks by direct reaction with DNA [61,62]. However, single strand breaks may be transformed into double strand breaks by enzymatic processing when damage is in close proximity or encountered by replication fork [61,62]. This may explain the double strand breaks occurred after 8 h of incubation with 2-ME while single strand breaks were observed as soon as after 2 h [12]. Nonetheless, the maximal induction of nitro-oxidative stress by 2-ME after 8 h of incubation what strictly correlated with double strand breaks after treatment with 2-ME [12].

In this study, we also aimed to complete the mechanism of induction and activation of nNOS by 2-ME. Interestingly, we observed that 2-ME at both pharmacological and physiological relevant concentrations resulted in a short-term transient activation of NF-κB p65. The mammalian NF-κB family consists of 5 protein members: NF-κB1 (p50 and its precursor p105), NF-κB2 (p52 and its precursor p100), RelA (p65), RelB, and c-Rel [[63], [64], [65]]. NF-kB transcriptional factor, as an inactive complex with inhibitory proteins called I-κB in the cytoplasm in normal resting cells, however, it can be activated to enter the nucleus where it regulates the expression of diverse genes controlling e.g. cell proliferation and cell death [[63], [64], [65]]. NF-Kb is well known anti-apoptotic factor, but it is also an important protein in response to cellular stress that can induce apoptosis [[63], [64], [65]] e.g., activation of the NF-κB pathway is essential for p53-mediated cell death [65]. Aberrant activation of NF-κB has been linked to inflammatory and autoimmune diseases, infection and cancer. Notably, NF-κB is constitutively activated in various malignancies such as lymphoma, gastrointestinal tumor, genitourinary, gynecological, thoracic, head, and neck tumours [66,67]. In addition, down-regulation of NF-κB attenuates proliferation, migration, and bone resorption in OS and the OS-microenvironment [[68], [69], [70]]. Therefore, given the tumor promoting role of NF-κB, targeting NF-κB for tumor prevention and therapy might be beneficial [66,67].

On the other hand, depending on the type of drug or cancer, activation of NF-κB can elicit a pro-death response [68]. Indeed, NF-κB is also activated by most chemotherapeutic agents and radiation used for the treatment of cancer [63]. There is evidence that NF-κB activation can sensitize cells to apoptosis and that inhibition of NF-κB results in resistance to chemotherapy [68]. While, in some cases, NF-κB activation by chemotherapeutic drugs elicits a pro-survival cellular response and combination with inhibitors of NF-κB improves efficacy [71]. Thus, the NF-κB may be considered as a double edge sword. 2-ME was previously reported to downregulate [72] or activate NF-Kb [71] signalling pathway depending on used experimental model.

The role of RNS in activation or de-activation of NF-Kb signalling pathway is also contradictory and seems to be dependent on the experimental models [73,74]. However, previous studies have clearly suggested that NF-κB might be involved in the upregulation of nNOS [[75], [76], [77]] in neural tissues. It was further reported that activation of NF-κB in retina cells results in the increased expression of nNOS, which subsequently leads to elevated production of ˙NO [78]. This enhanced production of ˙NO in turn causes the death of retina cells [78]. Notably, inhibitors of nNOS and NF-κB significantly reduced nNOS overexpression and ˙NO production in rat small intenstine model [79]. In cultured rat aortic smooth muscle cells, treatment with atorvastatin significantly increased nNOS expression, associated with activation of AKT and NF-κB [80].

Unfortunately, up to date the data considering role of NF-κB in regulation of nNOS in cancer cells are limited. Notably, acetylation of NF-kB p65 and p50 subunits by trichostatin A (TSA), a histone deacetylase inhibitor, augments their DNA binding affinities, thereby activating the nNOS exon 1f promoter in neuroblastoma cells [81]. Importantly, it was also proved that recoupling NOS inhibits NF-κB promoter activity [75]. Increasing BH_4_:BH_2_ may also results in decreased tyrosine nitration of IKBα but increased S-nitrosylation of the p65 subunit of NF-κB [19]. Recently, it was demonstrated that depletion of BH_4_ inhibits NF-κB activity [82].

Taking into consideration the role of NF-κb signalling pathway in anticancer effects of 2-ME needs to be further evaluated. However, we propose that short-term transient activation of NF-κb observed in OS experimental model is strictly associated with induction, activation of nNOS and local induction of nitro-oxidative stress, finally, resulting in cancer cell death.

## 5 Conclusions

Herein, we evidenced that ONOO^−^, its protonated form, ONOOH, and ^•^NO_2_ molecules, that production is at least partially dependent on selective nNOS overexpression, are involved in anticancer signalling of 2-ME due to short-term, transient NF*κ*B activation. Though the mechanism of 2-ME action is still undefined, the drug is a potent chemotherapeutic agent targeting actively proliferating cells with no cytotoxic effect to quiescent cells [[3], [4], [5], [6], [7], [8], [9], [10], [11], [12], [13], [14],^83^].

Importantly, observed effects like RNS production and short-term transient impact on NF*κ*B were induced by 2-ME also at physiological relevant concentrations. It may suggest that 2-ME may be considered as an anticancer physiological compound and a new hormone, what needs to be further studied.

**Fig. 5:**
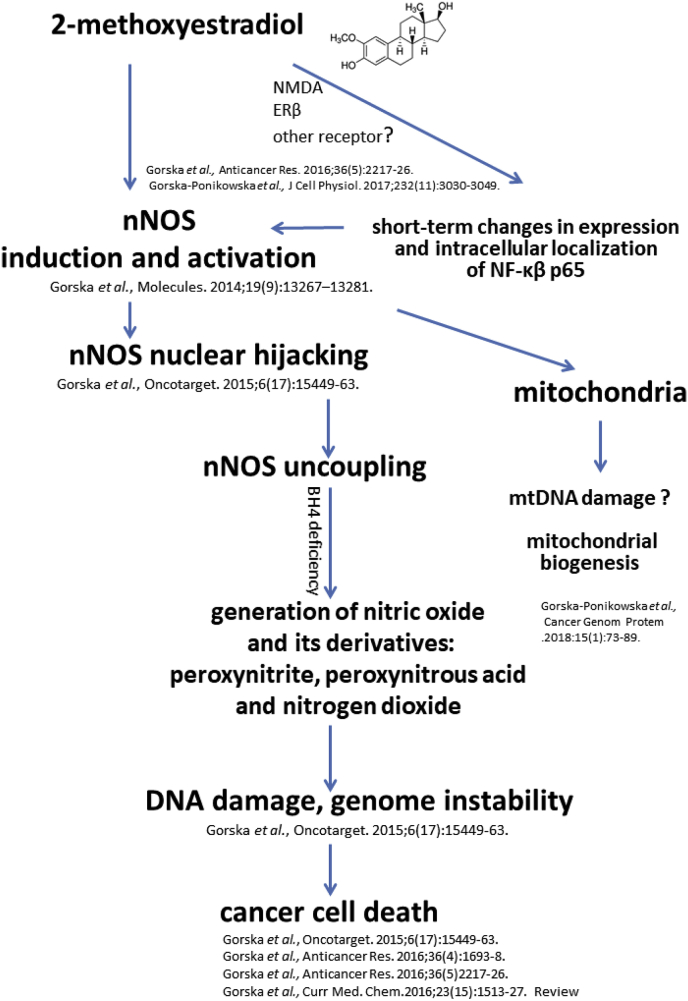
Graphical abstract presenting anticancer mechanisms of action of 2-ME relying on nNOS nuclear hijacking and enzyme uncoupling.

The anticancer activity of 2-ME may be sum up as presented in Fig. 5.

## Funding

Part of the studies and manuscript publication were supported by Iuventus Plus Project of Polish Ministry of Science and Higher Education No IP2015 022074.

Studies were partially supported by Ministry of Science and Higher Education Poland (grant ST46, MW), (grants DIR/WK/2017/01 and N N401 633640, AP, LWD, LK).

## Data availability

The data used to support the findings of this study are available from the corresponding author upon request.

## Declaration of competing interest

The authors declare no conflict of interest.

## The abbreviations

2-ME: 2-Methoxyestradiol
RNS: Reactive Nitrogen Species
NOS: Nitric Oxide Synthase
nNOS: neuronal Nitric Oxide Synthase
eNOS: endothelial Nitric Oxide Synthase
iNOS: inducible Nitric Oxide Synthase
L-NDBA: Nω-Nitroarginine-2,4-l-diaminobutyric amide di(trifluoroacetate)
PBS: Phosphate Buffered Saline.
